# Musical neurofeedback for treating depression in elderly people

**DOI:** 10.3389/fnins.2015.00354

**Published:** 2015-10-02

**Authors:** Rafael Ramirez, Manel Palencia-Lefler, Sergio Giraldo, Zacharias Vamvakousis

**Affiliations:** ^1^Department of Information and Communication Technologies, Universitat Pompeu FabraBarcelona, Spain; ^2^Department of Communication, Universitat Pompeu FabraBarcelona, Spain

**Keywords:** music, neurofeedback, emotions, expressive performance, depression, electroencephalography, elderly patients

## Abstract

We introduce a new neurofeedback approach, which allows users to manipulate expressive parameters in music performances using their emotional state, and we present the results of a pilot clinical experiment applying the approach to alleviate depression in elderly people. Ten adults (9 female and 1 male, mean = 84, SD = 5.8) with normal hearing participated in the neurofeedback study consisting of 10 sessions (2 sessions per week) of 15 min each. EEG data was acquired using the Emotiv EPOC EEG device. In all sessions, subjects were asked to sit in a comfortable chair facing two loudspeakers, to close their eyes, and to avoid moving during the experiment. Participants listened to music pieces preselected according to their music preferences, and were encouraged to increase the loudness and tempo of the pieces, based on their *arousal* and *valence* levels. The neurofeedback system was tuned so that increased arousal, computed as beta to alpha activity ratio in the frontal cortex corresponded to increased loudness, and increased *valence*, computed as relative frontal alpha activity in the right lobe compared to the left lobe, corresponded to increased tempo. Pre and post evaluation of six participants was performed using the BDI depression test, showing an average improvement of 17.2% (1.3) in their BDI scores at the end of the study. In addition, an analysis of the collected EEG data of the participants showed a significant decrease of relative alpha activity in their left frontal lobe (*p* = 0.00008), which may be interpreted as an improvement of their depression condition.

## Introduction

There is ample literature reporting on the importance and benefits of music for older adults (Ruud, 1997; Cohen et al., 2002; McCaffrey, 2008). Some studies suggest that music contributes to positive aging by promoting self-esteem, feelings of competence and independence while diminishing the feelings of isolation (Hays and Minichiello, 2005). Listening to music appears to be rated as a very pleasant experience by older adults since it promotes relaxation, decreases anxiety, and distracts people from unpleasant experiences (Cutshall et al., 2007; Ziv et al., 2007; Fukui and Toyoshima, 2008). It can also evoke very strong feelings, both positive and negative, which very often result in physiological changes (Lundqvist et al., 2009). These positive effects seem to be also experienced by people with dementia (Särkämö et al., 2012, 2014; Hsu et al., 2015). All these findings have led many researchers to be interested in the topic of the contribution of music to the quality of life and to life satisfaction of older people (Vanderak et al., 1983). Music activities (both passive and active) can affect older adults' perceptions of their quality of life, valuing highly the non-musical dimensions of being involved in music activities such as physical, psychological, and social aspects (Coffman, 2002; Cohen-Mansfield et al., 2011). Music experiences, led by music therapists or by other caregivers, besides being a source of entertainment, seem to provide older people the mentioned benefits (Hays and Minichiello, 2005; Solé et al., 2010). Music has been shown to be beneficial in patients with different medical conditions. Särkämö et al. (2010) demonstrated that stroke patients merely listening to music and speech after neural damage can induce long-term plastic changes in early sensory processing, which, in turn, may facilitate the recovery of higher cognitive functions. The Cochrane review by Maratos et al. (2008) highlighted the potential benefits of music therapy for improving mood in those with depression. Erkkilä et al. (2011) showed that music therapy combined with standard care is effective for depression among working-age people with depression. In their study, patients receiving music therapy plus standard care showed greater improvement in depression symptoms than those receiving standard care only.

Neurofeedback has been found to be effective in producing significant improvements in medical conditions such as depression (Kumano et al., 1996; Rosenfeld, 2000; Hammond, 2004), anxiety (Vanathy et al., 1998; Kerson et al., 2009), migraine (Walker, 2011), epilepsy (Swingle, 1998), attention deficit/hyperactivity disorder (Moriyama et al., 2012), alcoholism/substance abuse (Peniston and Kulkosky, 1990), and chronic pain (Jensen et al., 2007), among many others (Kropotov, 2009). For instance, Sterman (2000) reports that 82% of the most severe, uncontrolled epileptics demonstrated a significant reduction in seizure frequency, with an average of a 70% reduction in seizures. The benefits of neurofeedback in this context were shown to lead to significant normalization of brain activity even when patients were asleep. The effectiveness of neurofeedback was validated compared to medication and placebo (Kotchoubey et al., 2001). Similarly, Monastra et al.'s (2002) research found neurofeedback to be significantly more effective than ritalin in changing ADD/ADHD, without having to remain on drugs. Other studies (Fuchs et al., 2003) have found comparable improvements with 20 h of neurofeedback training (forty 30-min sessions) to those produced by ritalin, even after only twenty 30-min sessions of neurofeedback (Rossiter and La Vaque, 1995). In the context of depression treatment, there are several clinical protocols used to apply neurofeedback such as shifting the alpha predominance in the left hemisphere to the right by decreasing left-hemispheric alpha activity, or increasing right hemispheric alpha activity, shifting an asymmetry index toward the right in order to rebalance activation levels in favor of the left hemisphere, and the reduction of Theta activity (4–8 Hz) in relation to Beta (15–28 Hz) in the left prefrontal cortex (i.e., decrease in the Theta/Beta ratio on the left prefrontal cortex) (Gruzelier and Egner, 2005; Michael et al., 2005; Ali et al., 2015). Dias and van Deusen (2011) applied a neurofeedback protocol that is simultaneously capable of providing the training demands of Alpha asymmetry and increased Beta/Theta relationship in the left prefrontal cortex.

A still relatively new field of research in affective computing attempts to detect emotion states in users using electroencephalogram (EEG) data (Chanel et al., 2006). Alpha and beta wave activity may be used in different ways for detecting emotional (arousal and valence) states of mind in humans. For instance, Choppin (2000) propose to use EEG signals for classifying six emotions using neural networks. Choppin's approach is based on emotional valence and arousal by characterizing valence, arousal and dominance from EEG signals. He characterizes positive emotions by a high frontal coherence in alpha, and high right parietal beta power. Higher arousal (excitation) is characterized by a higher beta power and coherence in the parietal lobe, plus lower alpha activity, while dominance (strength) of an emotion is characterized as an increase in the beta/alpha activity ratio in the frontal lobe, plus an increase in beta activity at the parietal lobe. Ramirez and Vamvakousis (2012) characterize emotional states by computing arousal levels as the prefrontal cortex beta to alpha ratio and valence levels as the alpha asymmetry between lobes. They show that by applying machine learning techniques (support vector machines with different kernels) to the computed arousal and valence values it is possible to classify the user emotion into high/low arousal and positive/negative valence emotional states, with average accuracies of 77.82, and 80.11%, respectively. These results show that the computed arousal and valence values indeed contain meaningful user's emotional information.

In this paper we investigate the potential benefits of combining music (therapy), neurofeedback and emotion detection for improving elderly people's mental health. Specifically, our main goal is to investigate the emotional reinforcement capacity of automatic music neurofeedback systems, and its effects for improving depression in elderly people. With this aim, we propose a new neurofeedback approach, which allows users to manipulate expressive parameters in music performances using their emotional state. The users' instantaneous emotional state is characterized by a coordinate in the arousal-valence plane decoded from their EEG activity. The resulting coordinate is then used to change expressive aspects of music such as tempo, dynamics, and articulation. We present the results of a pilot clinical experiment applying our neurofeedback approach to a group of 10 elderly people with depression.

## Materials and methods

### Participants

Ten adults (9 female and 1 male, mean = 84, SD = 5.8) with normal hearing participated in the neurofeedback study consisting of 10 sessions (2 sessions per week) of 15 min each. Participants granted their written consent and procedures were positively evaluated by the Clinical Research Ethical Committee of the Parc de Salut Mar (CEIC-Parc de Salut Mar), Barcelona, Spain, under the reference number: 2015/6343/I. EEG data was acquired using the Emotiv EPOC EEG device. Participants were either residents or day users in an elderly home in Barcelona and were selected according to their cognitive capacities, sensitivity to music and depression condition: all of them declared to regularly listen to music and presented with a primary complaint of depression, which was confirmed by the psychologist of the center. Informed consent was obtained from all participants. There were four people who abandoned the study toward the end of it due to illness.

### Materials

#### Music material

Prior to the first session, the participants in the study were interviewed in order to determine the music they liked and to identify particular pieces to be included in their feedback sessions. Following the interviews, for each participant a set of 5–6 music pieces was collected from commercial audio CDs. During each session a subset of the selected pieces was played to the participant. Table 1 shows the selected pieces for each participant.

**Table 1:** **Selected pieces for each participant**.

**Participant**	**Music pieces**
Subject 1	Por una cabeza (tango)/15 años tiene mi amor (Dúo Dinámico)/Memorias de África/Historia de un amor (Lucho Gatica)/La balanguera (Marina Rosell)/Maria (3 tenors)
Subject 2	Largo (Häendel)/Doctor Zhivago/Una rosa y una flor (Nino Bravo)/Claro de luna (Beethoven)/Abrázame (Julio Iglesias)/La leyenda del beso
Subject 3	Por una cabeza (tango)/Vals de las flores (Tchaikovsky)/La tabernera del puerto –zarzuela (3 tenores)/El lago de los cisnes (Tchaikovsky)/La balanguera (Marina Rosell)
Subject 4	Va pensiero (G.Verdi)/El meu avi (habanera)/Canción del ruiseñor –zarzuela Doña Francisquita/La sardana de les monges/El lago de los cisnes (Tchaikovsky)
Subject 5	Qué tiempo tan feliz (José Guardiola)/Mira que eres linda (A. Machin)/Paraules d'amor (Serrat)/Toda una vida/El día que me quieras (C.Gardel)/Angelitos negros (A.Machin)
Subject 6	La balanguera (Marina Rosell)/Amparito Roca (pasodoble)/El meu avi (habanera)/Paquito el chocolatero (pasodoble)/El Danubio azul (Strauss)
Subject 7	Concierto Piano n.1 (Tchaikovsky)/Olas del Danubio (Ivanovici)/Only you (The Platters)/Claro de luna (Beethoven)/Largo (Häendel)
Subject 8	Himno del amor (Francisco)/Vals d'Amélie/Torna Asurriento (3 tenores)/Vals de las flores (Tchaikovsky)/De qué hablas –habanera (Marina Rosell)
Subject 9	Mi gran amor (Nino Bravo)/Nessum Dorma -3 tenors (G.Puccini)/De qué hablas –habanera (Marina Rosell)/Himno del amor (Francisco)/Vals d'Amélie/Torna Asurriento (3 tenores)
Subject 10	Mira que eres linda (A. Machin)/Paquito el chocolatero (pasodoble)/Cambalache –tango (C.Gardel)/Perdón (Los Panchos)/Lacrimosa –Requiem (Mozart)/Aquellas pequeñas cosas (Serrat)

#### Data acquisition and processing

**Figure 1:**
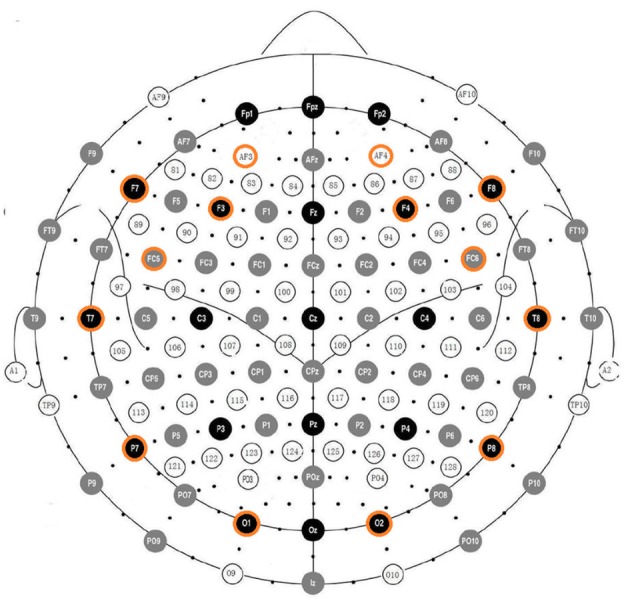
**Positions of the Emotiv EPOC electrodes aligned with positions in the 10–20 system**.

The Emotiv EPOC EEG system (Emotiv, 2014^1^) was used for acquiring the EEG data. It consists of 16 wet saline electrodes, providing 14 EEG channels, and a wireless amplifier. The electrodes were located at the positions AF3, F7, F3, FC5, T7, P7, O1, O2, P8, T8, FC6, F4, F8, AF4 according to the international 10–20 system (see Figure 1). Two electrodes located just above the subject's ears (P3, P4) were used as reference. The data were digitized using the embedded 16-bit ADC with 128 Hz sampling frequency per channel and sent to the computer via Bluetooth. The EEG signals were band-pass filtered with Butterworth 8–12 Hz and 12–28 Hz filters. The impedance of the electrode contact to the scalp was visually monitored using Emotiv Control Panel software.

The Emotiv EPOC EEG system is part of a number of low-cost EEG systems, which have been recently commercialized [a usability review of some of them can be found in (10)]. These systems are mainly marketed as gaming devices and the quality of the signal they capture is lower than the signal captured by more expensive equipment. However, recent research on evaluating the reliability of some of these low-cost EEG devices for research purposes has suggested that they are reliable for measuring EEG signals (Debener et al., 2012; Thie et al., 2012; Badcock et al., 2013). In the case of our study, the Emotiv EPOC device has provided several important pragmatic advantages compared with more expensive equipment: the setting up time of the Emotiv EPOC system at the beginning of each session is considerably shorter than that of an expensive EEG system (for which an experienced clinical professional can take up to an hour to place the electrodes on the patient's scalp, which results in long and tedious sessions). Furthermore, expensive EEG systems typically require the application of conductive gel in order to create a reliable connection between each electrode and the patient's scalp (the gel attaches to the patient's hair and can only be properly removed by washing the entire head at the end of each session). The setting up time of the Emotiv EPOC takes a few minutes (typically 3–5 min) and conductive gel is not necessary for the Emotiv EPOC's wet saline electrodes. However, the inferior signal quality of the Emotiv EPOC device is a limitation of this study, and thus it should be emphasized that future studies should involve the use of a more accurate EEG device.

We collected and processed the data using the OpenViBE platform (Renard et al., 2010). In order to play and transform the music feedback through the OpenVibe platform, a VRPN (Virtual-Reality Peripheral Network) to OSC (Open Sound Control protocol) gateway was implemented and used to communicate OpenViBE with Pure Data (Puckette, 1996). OSC is a protocol for networking sound synthesizers, computers, and other multimedia devices for purposes such as musical performance, while VRPN is a device-independent and network-transparent system for accessing virtual reality peripherals in applications. The VRPN-OSC-Gateway connects to a VRPN server, converts the tracking data and sends it to an OSC server. Music feedback was played by AudioMulch VST-host application, which received MIDI messages from Pure Data, and in which a *tempo transformation plugin* (Mayor et al., 2011) was installed. The plugin allows performing pitch-independent real-time tempo transformations (i.e., time stretch transformations) using audio spectral analysis-synthesis techniques. The plugin parameters were controlled using the MIDI messages sent by Pure Data. Music tempo and loudness were controlled assigning the corresponding MIDI control message coming from Pure Data. Both data acquisition and music playback were performed on a laptop with an Intel Core i5 2.53 Ghz processor with 4 GB of RAM, running windows 7 64-bit Operating System and using the laptop's internal sound card (Realtek ALC269). Music was amplified by two loudspeakers Roland MA150U.

### Methods

**Figure 2:**
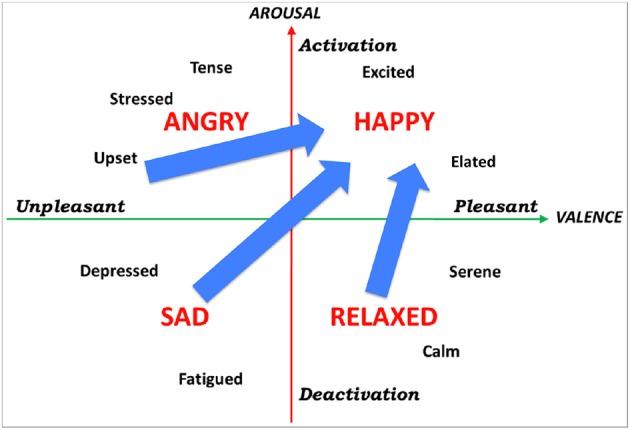
**Arousal-valence plane**. By encouraging participants to increase the loudness and tempo of musical pieces, they were encouraged to increase their arousal and valence, and thus direct their emotional state to the top-right quadrant in the arousal-valence plane.

Participants were treated individually. At the beginning of each feedback session, participants were informed about the experiment procedure, were asked to sit in a comfortable chair facing two loudspeakers, to close their eyes, and avoid moving during the experiment. Participants listened to preselected music pieces according to their music preferences for 15 min. Within these 15 min music pieces were separated by a pause of 1 s. Participants were encouraged to increase the loudness and tempo of the pieces so the pieces sounded “happier.” As the system was tuned so that increased *arousal* corresponded to increased loudness, and increased *valence* corresponded to increased tempo, participants were encouraged to increase their arousal and valence, in other words to direct their emotional state to the high-arousal/positive-valence quadrant in the arousal-valence plane (see Figure 2). At the end of each session, participants were asked if they perceived they were able to modify the music tempo and volume. Pre and post evaluation of participants was performed using the BDI depression test.

No artifact detection/elimination method was applied to the measured EEG signal. Both electrooculographic (EOG) and electromyographic (EMG) artifacts were minimized by asking participants to close their eyes and avoid movement. No control of the interface through eye or muscle movement was observed during the experimental sessions. However, it must be noted that it is important to extend/redo the reported study using artifact detection methods.

The EEG data processing was adapted from Ramirez and Vamvakousis (2012). Based on the EEG signal of a person, the arousal level was determined by computing the ratio of the beta (12–28 Hz) and alpha (8–12 Hz) brainwaves. EEG signal was measured in four locations (i.e., electrodes) in the prefrontal cortex: AF3, AF4, F3, and F4 (see Figure 1). Beta waves β are associated with an alert or excited state of mind, whereas alpha waves α are more dominant in a relaxed state. Alpha activity has also been associated to brain inactivation. Thus, the beta/alpha ratio is a reasonable indicator of the arousal state of a person. Concretely, arousal level was computed as following:

(1) $$\begin{array}{l} Arousal=(\beta F3+\beta F4+\beta AF3+\beta AF4)/(\alpha F3+\alpha F4+\alpha AF3\\ \;+\;\alpha AF4) \end{array}$$

In order to determine the valence level, activation levels of the two cortical hemispheres were compared. A large number of EEG studies (Henriques and Davidson, 1991; Davidson, 1992, 1995, 1998), have demonstrated that the left frontal area is associated with more positive affect and memories, and the right hemisphere is more involved in negative emotion. F3 and F4 are the most used positions for looking at this alpha/beta activity related to valence, as they are located in the prefrontal lobe, which plays a crucial role in emotion regulation and conscious experience. Valence values were computed by comparing the alpha power α in channels F3 and F4. Concretely, valence level was computed as following:

(2) Valence=αF4−αF3

Valence and arousal computation was adapted from Ramirez and Vamvakousis (2012), where the authors show that the computed arousal and valence values indeed contain meaningful user's emotional information.

**Figure 3:**
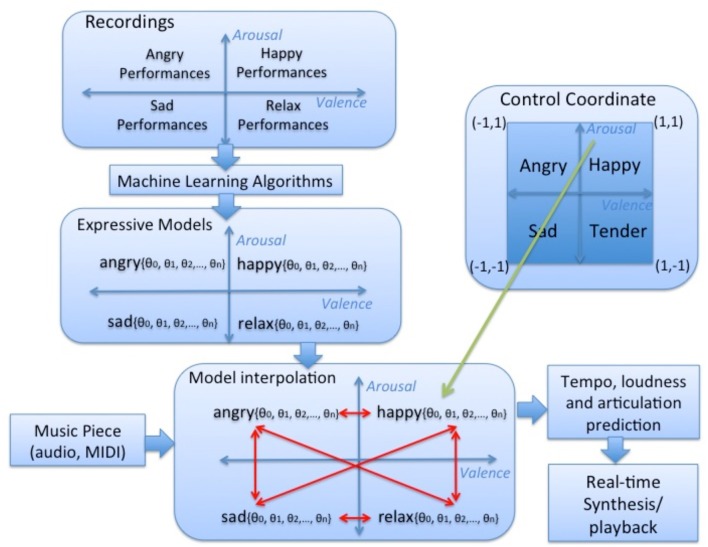
**Overview of the expressive music performance system**. Happy, relaxed, sad, and angry models were learnt from music recordings with these emotions using machine learning techniques and interpolated in order to obtain intermediate models and corresponding performance predictions.

**Figure 4:**
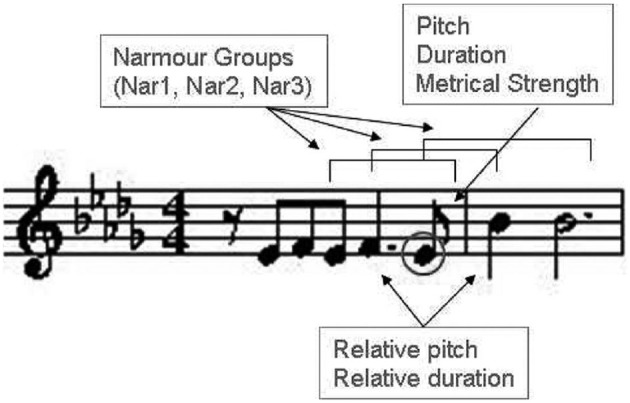
**Note characterization in performances**.

Computed arousal and valence values are fed into an expressive music performance system which calculates appropriate expressive transformations on timing, loudness and articulation (however, in the present study only timing and loudness transformations are considered). The expressive performance system is based on a music performance model, which was obtained by training four models using machine learning techniques (Mitchell, 1997) applied to recordings of musical pieces in four emotions: *happy, relaxed, sad*, and *angry* (each corresponding to a quadrant in the arousal-valence plane). The coefficients of the four models were interpolated in order to obtain intermediate models (in addition to the four trained models) and corresponding performance predictions (Figure 3). Details about the expressive music performance system and our approach to expressive performance modeling can be found in (Ramirez et al., 2010; Giraldo, 2012; Ramirez et al., 2012; Giraldo and Ramirez, 2013; Marchini et al., 2014). In order to model expression in music performances we characterized each performed note by a set of inter-note features representing both properties of the note itself and aspects of the musical context in which the note appears (Figure 4). Information about the note included note pitch (*Pitch*), note duration (*dur*), and note metrical strenght (*MetrStr*), while information about its melodic context included the relative pitch and duration of the neighboring notes (*PrevPitch, PrevDur, NextPitch, NextDur*), i.e., previous and following notes, as well as the music structure (i.e., Narmour groups) in which the note appears (Narmour, 1991). We also extracted the amount of legato with the previous note, the amount of legato with the following note, and mean energy. We applied machine learning techniques to train a linear regression models for predicting duration, and energy deviations expressed as a ratio of the values specified in the score (for energy which is not specified in the score we take the score value as the average of the energy of all the notes in the piece). For instance, for duration a predicted value of 1.14 represents a prediction of 14% lengthening of the note with respect to the score. In the case of energy it indicates that the note should be played a 14% louder than the average energy in the piece.

**Figure 5:**
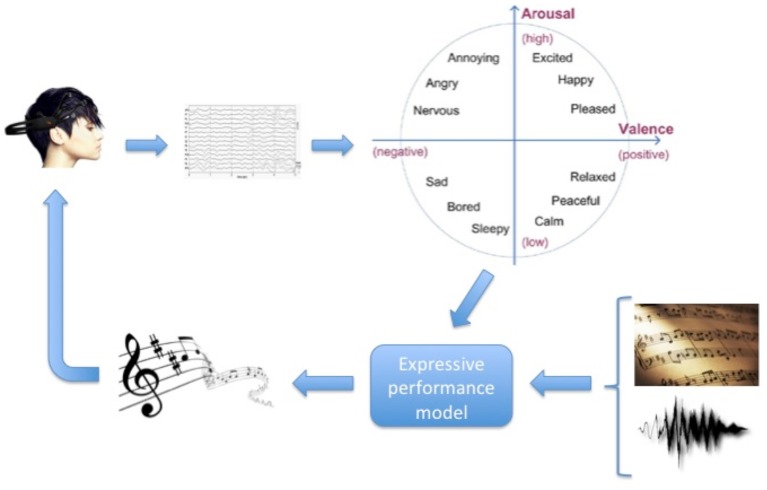
**Neurofeedback system Overview: real-time feedback loop in which the brain activity of a person is processed to produce an expressive rendition of a music piece according to the person's estimated emotional state**.

The general proposed emotion-based musical neurofeedback system is depicted in Figure 5. The system consisted of a real-time feedback loop in which the brain activity of participants was processed to estimate their emotional state, which in turn was used to control an expressive rendition of the music piece. The user's EEG activity is mapped into a coordinate in the arousal-valence space that is fed to a pre-trained expressive music model in order to trigger appropriate expressive transformations to a given music piece (audio or MIDI).

## Results

**Figure 6:**
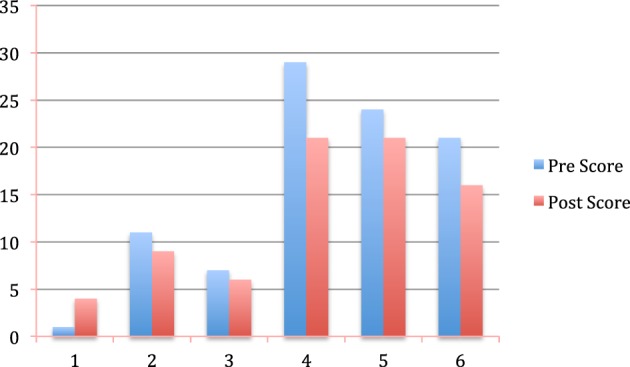
**Pre and post BDI depression test results for six participants**.

Seven participants completed training, requiring a total of ten 15 min sessions (2.5 h) of neurofeedback, with no other psychotherapy provided. There were four people who abandoned the study toward the end of it due to health problems. Pre and post evaluation of 6 participants was performed using the BDI depression test (One participant was not able to respond to the BDI depression test at the end of the treatment due to serious health reasons). The BDI evaluation performed using the BDI depression test, showed an average improvement of 17.2% (1.3) in BDI scores at the end of the study. Pre–post changes on the BDI test are shown in Figure 6.

We computed average valence and arousal values at the beginning of the first session and the beginning of the last session of the study. The obtained average valence values were 0.74 (0.22) and 0.83 (0.26) for the beginning of the first session and the beginning of the last session, respectively, while the obtained average arousal values were 0.97 (0.14) and 0.98 (0.21) for the beginning of the first session and the beginning of the last session, respectively (Table 2).

**Table 2:** **Arousal and valence values at the beginning and at the end of the study**.

**Indicators**	**Beginning**		**End**	
	**Average**	**SD**	**Average**	**SD**
Arousal	0.97	0.14	0.98	0.21
Valence	0.74	0.22	0.83	0.26

**Figure 7:**
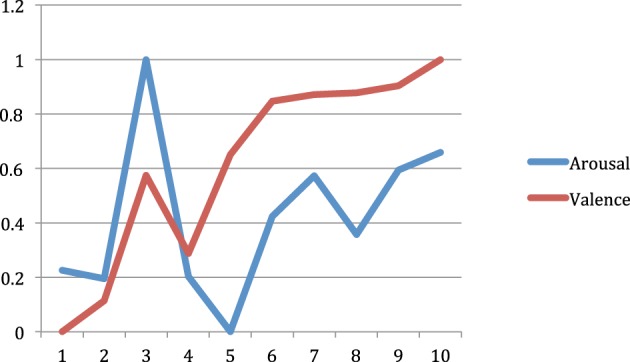
**Within session arousal and valence (normalized) values across ten 1 min periods**.

Figure 7 shows the correlation within sessions between the computed arousal and valence values, and time (1 min periods) within sessions. For valence we obtained a correlation of *r* = 0.919 (*p* = 0.000171) while for arousal we obtained a correlation of *r* = 0.315 (*p* = 0.375335).

## Discussion

Five out of six participants who responded to the BDI test made improvements in their BDI, and one patient improved from depressed to slight perturbation in the BDI scale. One participant, who initially scored as not depressed in the BDI pre-test (score = 1), did not show any improvement in her BDI post-score (score = 4), which was also in the non-depressed range. Either the participant was not depressed at the beginning of the study, or her responses to the BDI tests were not reliable. Excluding her from the BDI test analysis, the mean decrease in BDI scores was 20.6% (0.06). These differences were found significant (*p* = 0.018).

EEG data obtained during the course of the study showed that overall valence level increased at the end of the treatment compared to the starting level. The difference between valence values at the beginning and end of the study is statistically significant (*p* = 0.00008). This result should be interpreted as a decrease of relative alpha activity in the left frontal lobe, which may be interpreted as an improvement of the depression condition (Henriques and Davidson, 1991; Gotlib et al., 1999). Arousal values at the beginning and at the end of the study showed no significant difference (*p* = 0.33). However, in this study the most important indicator was valence since it reflects changes in negative/positive emotional states, which are directly related to depression conditions.

Correlation between valence values and time within sessions was found significant (*p* < 0.00018) but that was not the case for the correlation between arousal values and time. The fact that arousal-time correlation was not significant is not a negative result since valence is the most relevant indicator for depression.

Taking into account the obtained within- and cross-session improvements in valence levels and the limited duration of both each session (i.e., 15 min) and the complete treatment (i.e., 10 sessions), it may be reasonable to think that further improvements in valence could have been reached if sessions and/or treatment had been longer. We plan to investigate the impact of treatments with longer duration in the future.

Very few studies in the literature have examined the long-term effect of neurofeedback, but the few studies that did it found promising results (Gani et al., 2008; Gevensleben et al., 2010). Both Gani et al. (2008) and Gevensleben et al. (2010) showed that after the end of their studies, improvements were maintained and some additional benefits could be noted, suggesting that patients were still improving even after the end of treatment. In the current study, in addition to the post study BDI test, no follow-up for the participants was conducted in order to examine the long-term effect of our approach. This issue should be investigated in the future.

As it is the case of most of the literature on the use of neurofeedback to treat depression, which mainly represent uncontrolled case study reports, no control group has been considered in this pilot study. In order to quantify the benefits of combining music and neurofeedback compared to other approaches, ideally 3 groups should have been considered: one group with music therapy, one group with neurofeedback, and one group with the proposed approach combining music therapy and neurofeedback. In this way it would have been possible to quantify the added value of combining music therapy and neurofeedback. However, due to the limited number of participants this was not possible.

Some researchers have showed that listening to music regularly during the early stages of rehabilitation can aid the recovery maintaining attention, and preventing depressed and confused moods in stroke patients (Särkämö et al., 2008). Särkämö et al. conclude that in addition to these effects, music listening may also have general effects on brain plasticity, as the activation it causes in the brain is in both hemispheres, and more widely distributed than that caused by verbal material alone. In the current study, we propose a new neurofeedback approach, which combines emotion-driven neurofeedback with (active) music listening. In the light of the mentioned benefits of music listening/receptive music therapy (Grocke et al., 2007), it is reasonable to think that incorporating music listening in a neurofeedback setting can only improve the benefits of traditional neurofeedback systems. Furthermore, we argue that the combination of neurofeedback and receptive music therapy provides the benefits of both techniques, while eliminating potential drawbacks of each separate technique. When considered as separate methods, the advantages of receptive music therapy and neurofeedback are clear: they both provide a noninvasive method with a lack of contraindications. In addition, neurofeedback is oriented to encourage patients to self-regulate their brain activity in order to promote beneficial activity patterns, while receptive music therapy relies on the emotional therapeutic effects of listening to music. These positive properties of both techniques are clearly preserved by the proposed approach. On the other hand, neurofeedback procedures often can be tedious and consist of tasks involving visual or auditory feedback with little or no emotional content (e.g., moving a car on a computer screen). Furthermore, a drawback of neurofeedback is that it is based on traditional EEG-rhythms (e.g., theta, alpha, beta), which are functionally heterogeneous and individual (Hammond, 2010). Receptive music therapy methods are combined with the difficulty of selecting music material corresponding to the individual needs of the patient (MacDonald, 2013). These shortcomings are avoided by the proposed system: The system provides attractive feedback consisting of music material specially selected by the individual participants, and it is based on high-level descriptors (i.e., arousal and valence) representing the emotional state of users.

The results obtained in the current study seem to indicate that music has the potential to be a useful component in neurofeedback treatment. However, future research needs to explore the effect of individual responses' variables to music through direct experimental comparison. Future investigation of individual variables, such as music sensibility (e.g., music experience/familiarity) and the impact of depression severity, in addition to more stringent methodology, is required.

In summary, we have introduced a new neurofeedback approach, which allows users to manipulate expressive parameters in music performances using their emotional state, and presented the results of a neurofeedback clinical pilot study for treating depression in elderly people. The neurofeedback study consisted of 10 sessions (2 sessions per week) of 15 min each initially involving 10 participants from a residential home for the elderly in Barcelona. Participants were asked to listen to music pieces preselected by them according to their music preferences, and were encouraged to increase the loudness and tempo of the pieces, based on their arousal and valence levels, respectively: *arousal* was computed as beta to alpha activity ratio in the frontal cortex, and *valence* was computed as relative frontal alpha activity in the right lobe compared to the left lobe. Pre and post evaluation of 6 participants was performed using the BDI depression test, showing an average improvement of 17.2% (1.3) in their BDI scores at the end of the study. Analysis of the participants' EEG data showed a decrease of relative alpha activity in their left frontal lobe, which may be interpreted as an improvement of their depression condition. The positive results of our clinical experiment, suggest that new research with the proposed music neurofeedback approach is worthwhile.

### Conflict of interest statement

The authors declare that the research was conducted in the absence of any commercial or financial relationships that could be construed as a potential conflict of interest.

## Footnotes

^1^Emotiv Systems Inc. Researchers. (2014). Available online at: http://www.emotiv.com/researchers/
